# Discrete Quadruple Stacks Formed in a Nanosized Metallorectangle

**DOI:** 10.1021/acs.inorgchem.4c02653

**Published:** 2024-08-10

**Authors:** Susana Ibáñez, Eduardo Peris

**Affiliations:** Institute of Advanced Materials (INAM), Centro de Innovación en Química Avanzada (ORFEO−CINQA), Universitat Jaume I, Av. Vicente Sos Baynat s/n, Castellón E-12006, Spain

## Abstract

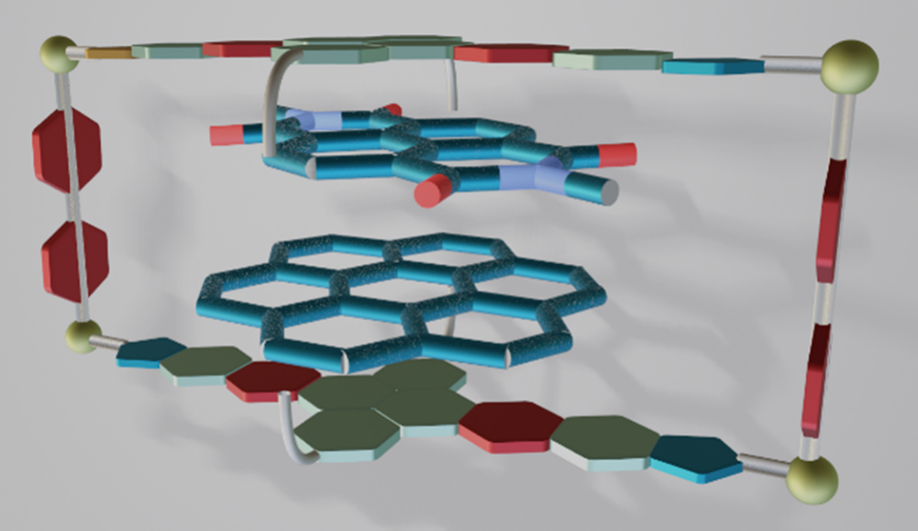

An iridium-cornered nanosized metallorectangle was obtained by combining a quinoxalinophenanthrophenazine-connected Janus-di-imidazolylidene ligand and 4,4′-bipyridine. This metallorectangle was used as host for a series of planar molecules, including pyrene, triphenylene, perylene, coronene, and N,*N*′-dimethyl-naphthalenetetracarboxy-diimide (NTCDI). The binding of coronene and NTCDI followed a strongly positive cooperative 1:2 stoichiometric binding model, as the inclusion of the first guest generates the geometrical requirements for the optimum encapsulation of the second planar molecule. The simultaneous encapsulation of coronene and NTCDI produces a heteroguest inclusion system, whose exchange dynamics was studied by means of variable temperature ^1^H NMR spectroscopy.

## Abstract 2

An iridium-conjoined nanosized metallorectangle is used for engineering quadruple stacks.

## Introduction

Supramolecular structures with the ability to bind different exogenous guests are ubiquitously present in nature.^1^ Supramolecular self-assembly mimicks nature’s principles for building complex and well-organized structures from simple building blocks.^2^ By following basic geometric rules, the combination of organic linkers and metal ions can lead to the programmed design of metallosupramolecular assemblies with a very high level of certainty.^2,3^ The resulting coordination cages possess binding pockets whose properties are determined by the overall geometry of the metal–organic complex.^4^ For the design of these pockets, the ligand is arguably the most important component, because its topological features and binding abilities determine the size, shape and functionality of the resulting cavities. In general, a good size and shape match between the cavity of the metallobox and the substrate is critical for promoting the association. The study of how multiple guests can bind to a single receptor can provide important information about new modes of host–guest interactions that may be translated into new applications,^4^ yet the selective inclusion of two different guests within a single host remains a very difficult task. In general, the length of the pillar ligand can be used to control the number of planar guest molecules than can be stacked within the cavity of the metallobox.^5^ One of the greatest challenges of supramolecular chemistry is to enable synthetic molecular receptors in which the binding of one substrate cooperatively affects the binding of the subsequent guest molecules, and can promote allosteric communication between all components of the supramolecular assembly. In particular, receptors that are capable of encapsulating multiple planar polyaromatic molecules that can form discrete stacks are particularly interesting, because enabling discrete π-stacks can facilitate the study of the charge transport at the molecular level, a critical issue for the design of nanoscale electronic devices.^5a−5f^

Poly-N-heterocyclic carbene ligands (NHCs) have recently appeared as very useful blocks for the construction of supramolecular organometallic complexes^6^ and, in particular, Janus-di-NHC ligands^7^ bridged by polycyclic aromatic hydrocarbons have shown interesting features regarding their host–guest chemistry properties.^8^

**Scheme 1:**
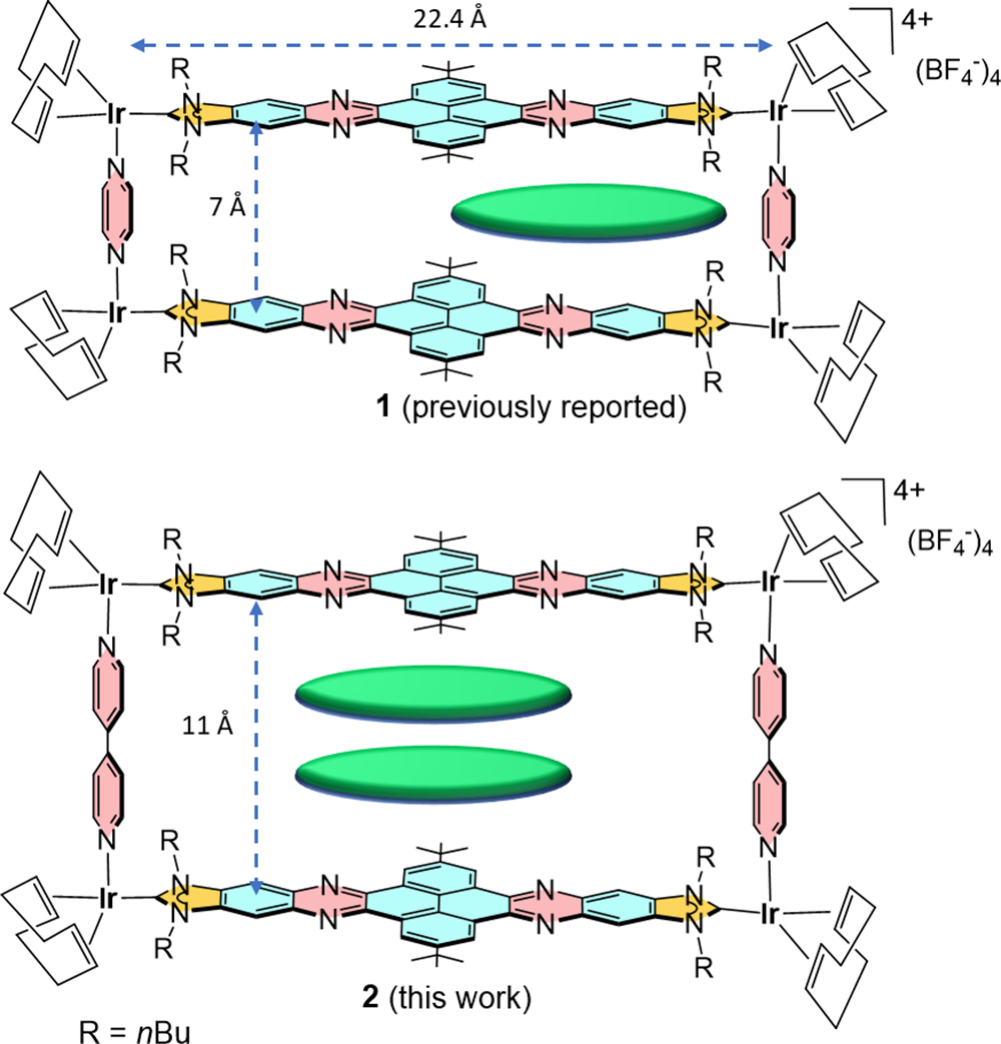
Two Metalloboxes Built using a Nanosized Janus-di-imidazolylidene Ligand

By using a 2.4 nm-long Janus di-NHC ligand, we recently described an iridium(I)-based metallorectangle that showed very large binding affinities with large planar polyaromatic molecules (**1**, in Scheme 1).^9^ An interesting feature of this long and narrow metallobox is that it displayed a large amplitude motion of the guests all along the long length of its cavity, thus behaving as a very unusual type of a molecular shuttle. Built on these grounds, we now describe the preparation of the tetra-iridium metallobox **2** (Scheme 1), obtained by combining the same quinoxalinophenanthrophenazine-connected Janus-di-imidazolylidene ligand with 4,4′-bipyridine. The utilization of 4,4′-bipyridine instead of pyrazine, allows establishing a distance of 11 Å between the polyaromatic panels of the di-NHC ligand, thus enabling the encapsulation of two planar polyaromatic guests. In this work we examined the host–guest chemistry properties of **2** with a series of polycyclic aromatic hydrocarbons. In addition, we also showed how **2** is able to accommodate two different aromatic guests.

## Results and Discussion

**Scheme 2:**
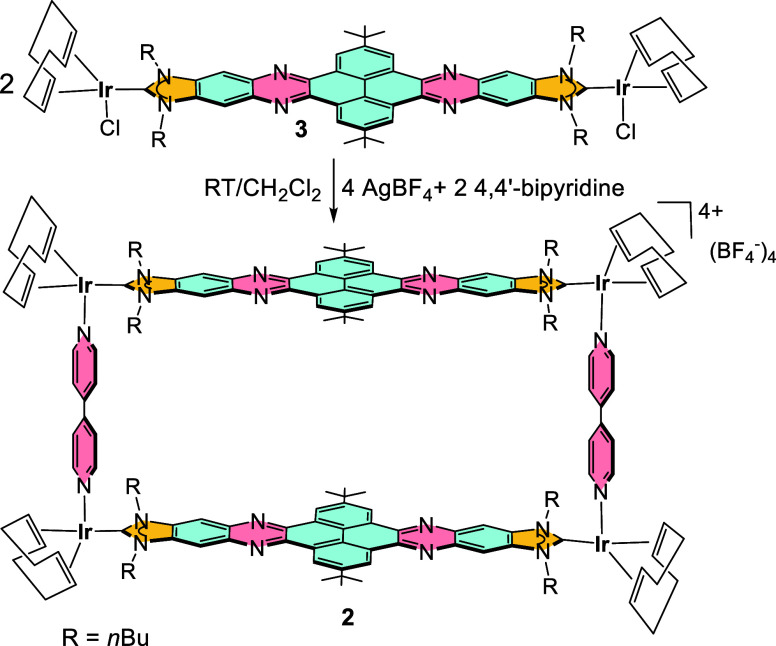
Synthesis of Metallorectangle **2**

The reaction between quinoxalinophenanthrophenazine-bis-imidazolylidene di-iridium(I) complex **3**(10) with 4,4′-bipyridine, in the presence of two equivalents of AgBF_4_ in CH_2_Cl_2_ leads to the air stable metallorectangle **2** in 81% yield, as it is shown in Scheme 2. Complex **2** was characterized by NMR, UV–vis and fluorescence spectroscopy, and gave satisfactory elemental analysis. The diffusion ordered NMR spectrum of **2** shows that all proton resonances display the same diffusion coefficient (4.67 × 10^–10^ m^2^/s in CD_2_Cl_2_), therefore indicating that all resonances belong to a single assembly. In addition, the diffusion coefficient of **2** is significantly smaller than that obtained for the smaller metallobox **1** (5.58 × 10^–10^ m^2^/s), as should be expected for a metallosupramolecular assembly with a larger hydrodynamic diameter. Unfortunately, the mass analysis of the complex via electrospray mass spectrometry (ESI-MS) allowed detection of only fragment ions generated from the thermal decomposition of the molecule, due to the relatively weak Ir(I)–N bonds.^11^

Then, we carried out a series of host–guest chemistry studies by performing a series of ^1^H NMR titrations using **2**, and a series of planar polycyclic aromatic hydrocarbons (PAHs) in CD_2_Cl_2_. The PAHs used were pyrene, triphenylene, perylene, coronene and N,*N*′-dimethyl-naphthalenetetracarboxy-diimide (NTCDI). The perturbations observed on the resulting ^1^H NMR spectra of **2** with the different guests, were highly dependent on the size of the guest used. In general, for all the guests used, we observed that the titrations induced the upfield shift of the two resonances due to the hydrogens of the polyaromatic linker of the di-NHC ligand, together with the downfield shift of the signals due to the protons of the bipyridine pillars. This observation indicates that the guests occupy the cavity of the metallorectangle, as this is the only region where the protons of the di-NHC ligands and bipyridines can be perturbed simultaneously. Another interesting observation is that the largest perturbations were observed for the resonances due to the protons of the central pyrene moieties of the host, thus suggesting that the interaction between the host and the guests is mainly produced in the central region of the cavity, in clear contrast with what we observed for the narrow metallobox **1** (Scheme 1), for which the guest is accommodated on one of the sides of the cavity, close to one of the pyrazine pillars.^9^ All titrations showed that the exchange process shows fast kinetics on the NMR time scale, as only averaged broad resonances of free host + guests, and guest@host complexes were observed. We also tried to obtain information about the host–guest binding affinities form the ^1^H NMR titrations. For the case of pyrene, triphenylene and perylene, we found it difficult to assess the stoichiometric binding model, as the relatively small perturbations found on the NMR titrations did not allow us to see clear differences when fitting the binding isotherms to 1:1 or 1:2 host–guest models. For the case of the titrations with coronene and NTCDI, by using a global nonlinear regression analysis,^12^ we observed that all titrations were best fitted to a 1:2 stoichiometry binding model. As we will discuss below, the 1:2 binding model was also supported by analyzing the ^1^H NMR spectra, which at low temperatures showed slow exchange kinetics on the NMR time scale (vide infra) and thus allowed the accurate calculation of the number of guests bound to the metallobox in solution. Both NMR titrations resulted in sigmoidal binding isotherms, which are consistent with strong positive cooperativity.^13^ For the case of coronene, we found that the binding constants were *K*_11_ = 14 ± 2 M^–1^, and *K*_12_ = (5.4 ± 0.9) × 10^3^ M^–1^. The constants associated with the binding with NTCDI were *K*_11_ = 73 ± 30 M^–1^, and *K*_12_ = (8.00 ± 0.13) × 10^3^ M^–1^. The larger binding constants shown by NTCDI compared to those shown by coronene, may be explained as a consequence of its electron-deficient nature (A), which forms more stable D–A–A–D stacks with the electron-rich (D) pyrene panels of the host, compared to the D–D–D–D stacks formed when coronene is used as guest. The positive cooperativity found for the binding of the two guests in **2** can be related to the positive cooperativity observed for one-dimensional one-component homogeneous stacks.^14^ In our case, the binding of the first molecule of guest produces only one face-to-face interaction between the polyaromatic panel of the host and one of the faces of the guest, thus producing a relatively small binding constant. The encapsulation of the second guest increases the number of face-to-face overlaps to three, as quadruple stacks of the type D–D–D–D (for coronene) or D–A–A–D (for NTCDI) are formed.

**Figure 1:**
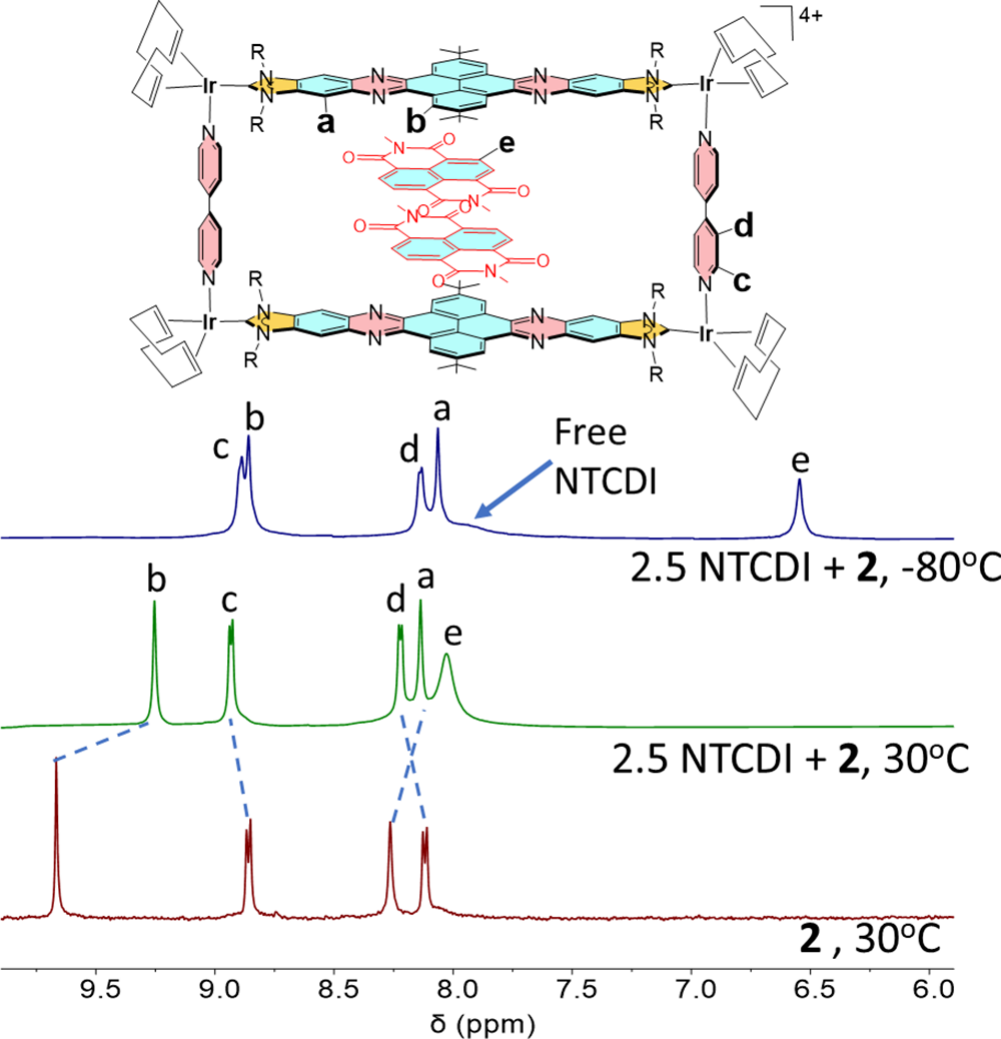
From bottom to top: aromatic region of the ^1^H NMR (500 MHz) spectra (CD_2_Cl_2_) of metallobox 2 at 30 °C; 2.5 eq of NTCDI+ 1 eq of metallobox **2** at 30 °C; and 2.5 eq. of NTCDI+ 1 eq of metallobox **2** at −80 °C. Blue dotted lines are used to signal the shift of the resonances due to free metallobox **2** upon addition of NTCDI.

As mentioned above, the ^1^H NMR spectra of mixtures of the planar polyaromatic guests with **2** showed fast kinetics on the NMR time scale at room temperature. In order to see if we could obtain information about the dynamic process, and about the orientation of the guest in the cavity of the metallobox, variable temperature ^1^H NMR spectroscopic experiments were performed in CD_2_Cl_2_ using 1:2.5 host/guest mixtures of **2** with coronene and NTCDI (see SI for full details). Figure 1 shows the ^1^H NMR spectra of the resulting solutions NTCDI and **2** at 30 and −80 °C, together with the spectrum of the free metallobox **2** for comparison. As can be observed from the spectra, the addition of 2.5 equiv of NTCDI at 30 °C results in the upfield shift of the resonances due to the hydrogens of the polyaromatic linker of the di-NHC ligand (a, b), together with the downfield shift of the signals due to the bipyridine ligands (c, d). In addition, a broad signal due to the aromatic protons of the NTCDI is observed at 8.2 ppm. Upon lowering the temperature down to −80 °C, both resonances due to the aromatic protons of the di-NHC ligand (a, b) shift further upfield, and the signal due to the aromatic protons of the NTCDI guest sharpens and shifts down to 6.5 ppm. The spectrum at −80 °C also shows a broad signal at 8.3 ppm due to residual free NTCDI, therefore indicating that at this temperature, the kinetics of the free guest/host@guest exchange is slow on the NMR time scale. The experiment also allows to reach two conclusions. First, the number of encapsulated guests is 2, as the comparison of the integrals of the signals due to the guest and the host allow establishing an accurate 2:1 molar ration between NTCDI and **2**. And second, the pattern of the ^1^H NMR spectrum indicates that the NTCDI guests are located in the central part of the cavity, as this is the only region within the host in which the symmetry of the metallobox is not broken. The fact that the guests are mainly located at the central part of the cavity contrasts with our previous results using the long and shallow metallobox **1** (Scheme 1), in which the guests were located on one of the sides of the cavity.^9^ We think that the larger pillar length provided by the bipyridine ligands (11 Å) allows the host to be flexible enough to adopt a stable conformational configuration in which the two planar guests can be located in the region where a maximum π–π-stacking interaction with the pyrene moieties of the host, while avoiding the steric hindrance provided by the bulky *tert*-butyl groups.

**Figure 2:**
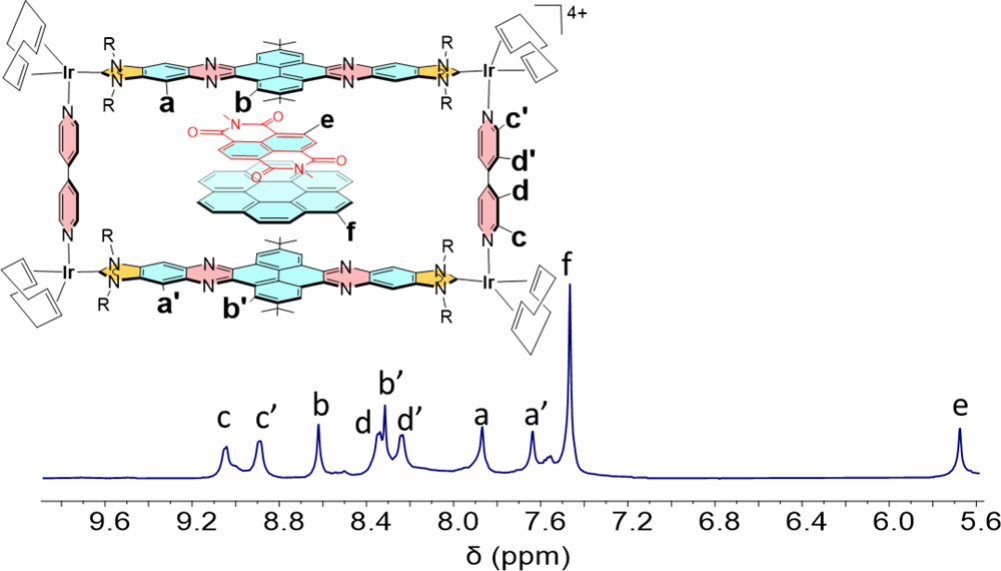
Aromatic region of the ^1^H NMR spectrum (500 MHz) of an equimolecular mixture (1 mM) of coronene, NTCDI, and **2** in CD_2_Cl_2_ at −80 °C.

**Figure 3:**
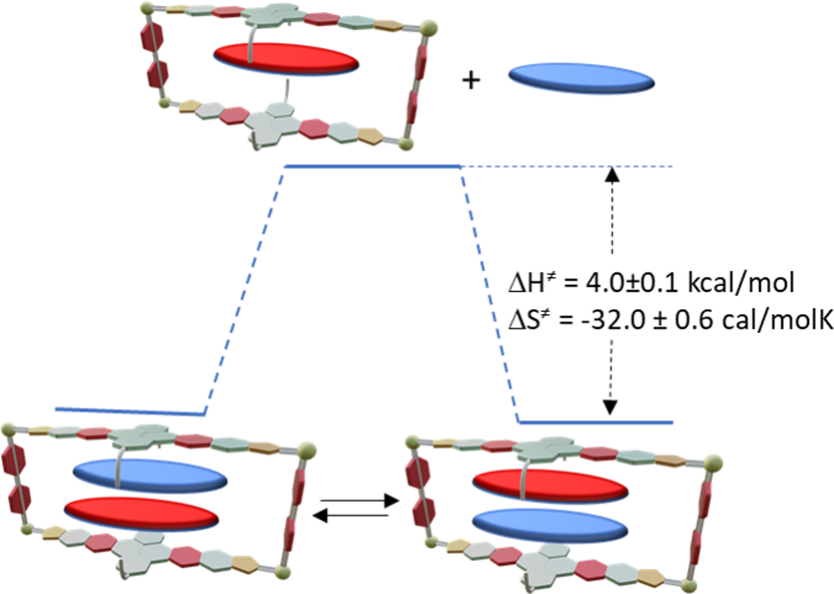
Schematic kinetic profile of the intermolecular dynamic exchange observed for (coronene)(NTCDI)@**2**. Blue disk represents coronene, and the red disk represents NTCDI.

We were also interested in exploring the simultaneous encapsulation of NTCDI and coronene in **2**. The ^1^H NMR spectrum of a solution containing an equimolecular amount of NTCDI, coronene and **2** in CD_2_Cl_2_, showed very broad signals suggestive of a dynamic exchange process whose kinetics is close to the NMR time scale. This dynamic exchange most likely involves the rapid site-exchange between coronene and NTCDI. Lowering the temperature down to −80 °C, allows obtaining a spectrum that is consistent with the loss of symmetry of the host upon encapsulation of the NTCDI-coronene pair. The low temperature spectrum shows eight resonances due to the aromatic protons of the metallobox, as should be expected for a dissymmetrical product consistent with (coronene)(NTCDI)@**2** (the presence of the heteroguests breaks the symmetry between the bottom and top part of the metallobox) (Figure 2). The loss of symmetry of the metallobox can also be used for studying the host–guest dynamics using VT-^1^H NMR measurements. From the line-shape analysis of the ^1^H NMR spectra, the activation barriers for the exchange process were estimated to be Δ*H*^‡^ = 4.0 ± 0.1 kcal/mol and Δ*S*^‡^ = −32.0 ± 0.6 cal/mol K (Figure S18). The enthalpy cost may arise from the initial dissociation of the weaker bound guest (most likely coronene). The large and negative entropy indicates that the transition state is more solvated than the ground state. This can be related with a mechanism of exchange involving the dissociation of the host–guest complex (Figure 3), as the dissociation of the guest is accompanied by the solvation of both, the host and the guest.

**Figure 4:**
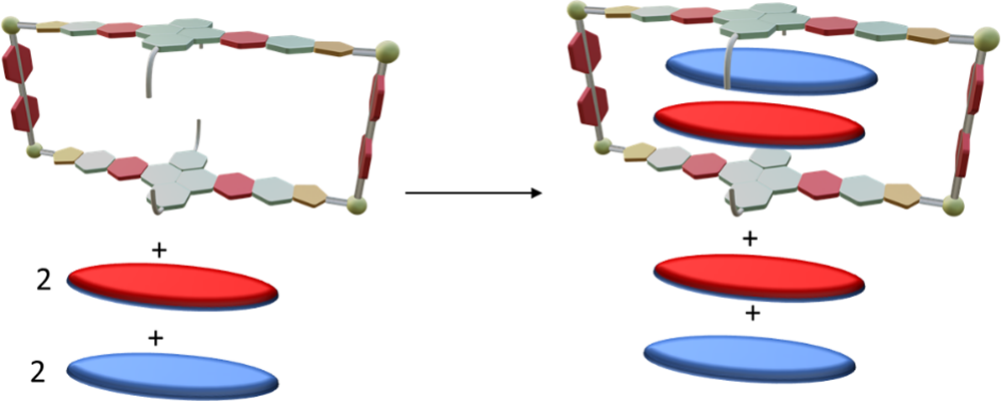
Selective formation of (coronene)(NTCDI)@**2**.

Finally, in order to compare the relative stability of (coronene)_2_@**2**, (NTCDI)_2_@**2** and (coronene)(NTCDI)@**2**, we analyzed a mixture containing the metallobox **2** with two equivalents of coronene and two equivalents of NTCDI in CD_2_Cl_2_, and observed the exclusive formation of the inclusion complex (coronene)(NTCDI)@**2** as shown in Figure 4 (see also Figure S19 in the Supporting Information file). The selective formation of (coronene)(NTCDI)@**2** under equilibrium conditions emphasizes the greater stability of the hetero D–A–D–D complex over either of the homoguest complexes.

## Conclusions

In summary, we prepared a nanosized metallobox for engineering quadruple stacks. The encapsulation of the planar polyaromatic molecules follows a 1:2 cooperative model, in which the inclusion of the first guest renders the optimum conditions for the encapsulation of a second planar molecule, therefore illustrating a clear case of positive cooperativity in an artificial receptor. We also described the formation of dissymmetrical quadruple stacks, by encapsulating two different planar guests (coronene and NTCDI) inside the metallobox, and unveiled the kinetics that control the dynamics of the exchange process. Enabling effective methods for achieving tailor-made aromatic stacks, and controlling the kinetic parameters that determine their mechanisms of exchange is necessary to enrich our ability to manipulate material properties at the supramolecular level.

## Experimental Section

### Materials and Methods

The quinoxalinophenanthrophenazine-bis-imidazolylidene di-iridium(I) complex **3**,^15^ and N,*N*′-dimethyl-naphthalenetetracarboxy diimide^16^ were prepared according to literature methods. All other reagents were used as received from commercial suppliers. NMR spectra were recorded on a Bruker 300 MHz or Bruker 400 MHz or Varian 500 MHz using CD_2_Cl_2_ or C_2_D_2_Cl_4_ as solvents. Elemental analyses were carried out on a TruSpec Micro Series. The BindFitv0.5 program was employed for the calculation of the association constants. UV/visible absorption spectra were recorded on a Varian Cary 300 BIO spectrophotometer using dichloromethane under ambient conditions. Emission spectra were recorded on a modular Horiba FluoroLog-3 spectrofluorometer employing degassed dichloromethane.

### Synthesis of the Metallobox 2

A mixture of the iridium complex **3** (80.30 mg, 0.054 mmol), AgBF_4_ (21.35 mg, 0.107 mmol), and bipyridine (8.56 mg, 0.054 mmol) in dry dichloromethane (10 mL) is stirred overnight at room temperature. The solution was filtered through a short pad of Celite and concentrated to almost dryness. It was added ether to precipitate a yellow solid. Yield: 75.90 mg (81%). Elemental analysis calcd (%) for C_160_H_188_N_20_Ir_4_B_4_F_16_.3H_2_O: C, 52.03; H, 5.20; N, 7.45. Found C, 51.05; H, 5.15; N, 7.45. ^1^H NMR (300 MHz, CD_2_Cl_2_): δ 9.58 (s, 8H, C*H*_pyr_), 8.78 (d, ^3^J_H–H_ = 6 Hz, 8H, C*H*_bipy_), 8.18 (s, 8H, C*H*_quino_), 8.04 (d, ^3^J_H–H_ = 6 Hz, 8H, C*H*_bipy_), 5.32–5.14 (m, 8H, NC*H*_2_CH_2_CH_2_CH_3_), 4.82–4.65 (m, 8H, NC*H*_2_CH_2_CH_2_CH_3_), 4.23 (br s, 8H, C*H*_COD_), 3.87 (br s, 8H, C*H*_COD_), 2.53–2.32 (m, 16H, NCH_2_C*H*_2_CH_2_CH_3_), 2.17–1.98 (m, 16H, NCH_2_CH_2_C*H*_2_CH_3_), 1.80–1.63 (m, 32H, C*H*_2 COD_), 1.56 (s, 36H, C(C*H*_3_)), 1.11 (t, ^3^J_H–H_ = 9 Hz, 24H, NCH_2_CH_2_CH_2_C*H*_3_). ^19^F{^1^H} NMR (282 MHz, CD_2_Cl_2_): −150.50 (s). ^13^C NMR (75 MHz, C_2_D_2_Cl_4_): δ 201.64 (Ir-*C*_carbene_), 151.59 (*C*H_bipy_), 144.48 (*C*_Ar_), 142.87 (*C*_Ar_), 138.43 (*C*_Ar_), 136.55 (*C*_Ar_), 129.03 (*C*_Ar_), 125.80 (*C*_Ar_), 125.36 (*C*H_bipy_), 124.81 (*C*H_pyr_), 108.52 (*C*H_quino_), 87.95 (*C*H_COD_), 66.32 (*C*H_COD_), 49.81 (N*C*H_2_CH_2_CH_2_CH_3_), 36.02 (*C*(CH_3_)_3_), 32.89 (NCH_2_*C*H_2_CH_2_CH_3_), 32.02 (C(*C*H_3_)_3_), 30.13 (*C*H_2 COD_), 20.95 (NCH_2_CH_2_*C*H_2_CH_3_), 14.33 (NCH_2_CH_2_CH_2_*C*H_3_).

## Supporting Information Available

Experimental details concerning the titration studies, data used for nonlinear fittings, and VT-NMR studies and all necessary NMR and UV–vis spectra (PDF)
