# Supplementary material for: Discrete Quadruple Stacks Formed in a Nanosized Metallorectangle
Source: Inorg Chem. 2024 Aug 10;63(34):16070–4. doi: 10.1021/acs.inorgchem.4c02653 (PMC11351171; doi:10.1021/acs.inorgchem.4c02653)
Supplement: Supplementary file 1 — ic4c02653_si_001.pdf [file ic4c02653_si_001.pdf]

**Supporting information for:**

# Discrete quadruple stacks formed in a nanosized metallorectangle

*Susana Ibáñez*[*a] *and Eduardo Peris*[*a]

[a]Institute of Advanced Materials (INAM). Centro de Innovación en Química Avanzada (ORFEO-CINQA). Universitat Jaume I. Av. Vicente Sos Baynat s/n. Castellón. E-12006. Spain.

| | |
|---|---|
| **1. Spectroscopic data** | **S1-S3** |
| 1.1. $^{1}H$, $^{19}F$, $^{13}C$, $^{1}H$-$^{13}C$ HSQC and DOSY NMR spectra of **2** in $CD_2Cl_2$ | S1-S3 |
| **2. Photophysical properties** | **S4** |
| 2.1. UV-visible absorption spectra | **S4** |
| 2.2. Emission spectra | **S4** |
| **3. $^{1}H$ NMR titration experiments** | **S5-S12** |
| **4. Variable-temperature $^{1}H$ NMR experiments** | **S13-S16** |

## 1. Spectroscopic data

### 1.1. $^{1}H$, $^{19}F$, $^{13}C$, $^{1}H$-$^{13}C$ HSQC and DOSY NMR spectra of **2** in $CD_2Cl_2$

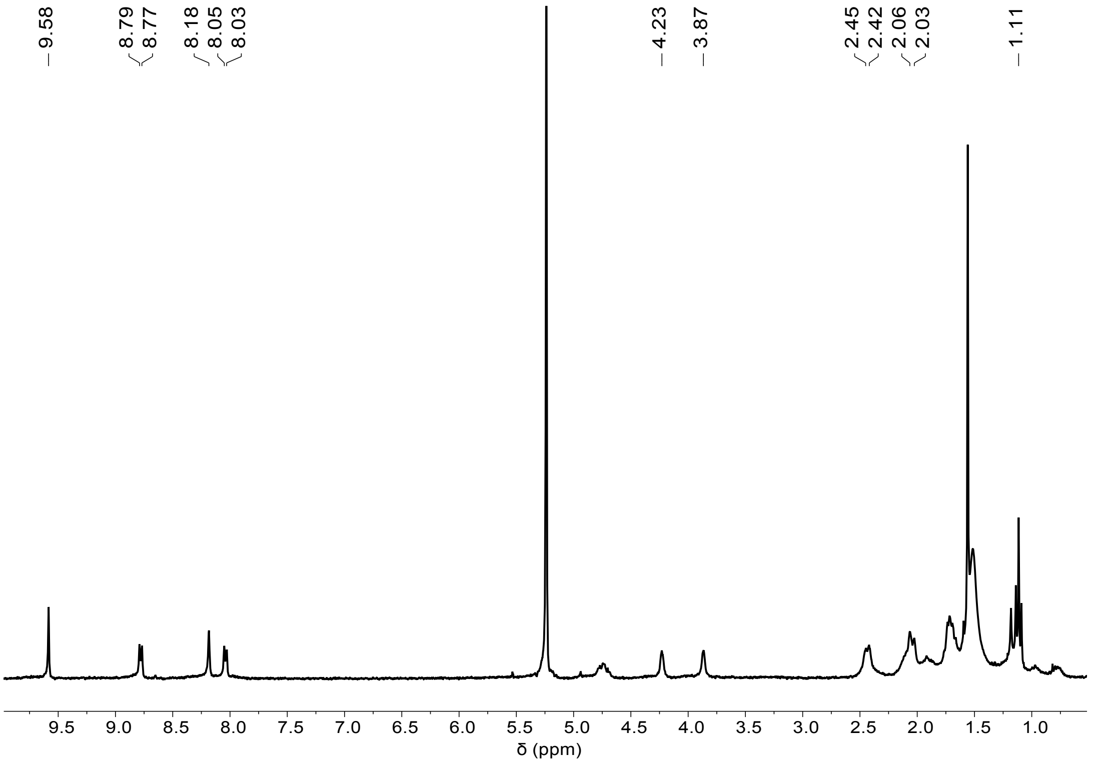


**Figure S1.** $^{1}H$ NMR spectrum (300 MHz) of **2** in $CD_2Cl_2$ at 298 K.

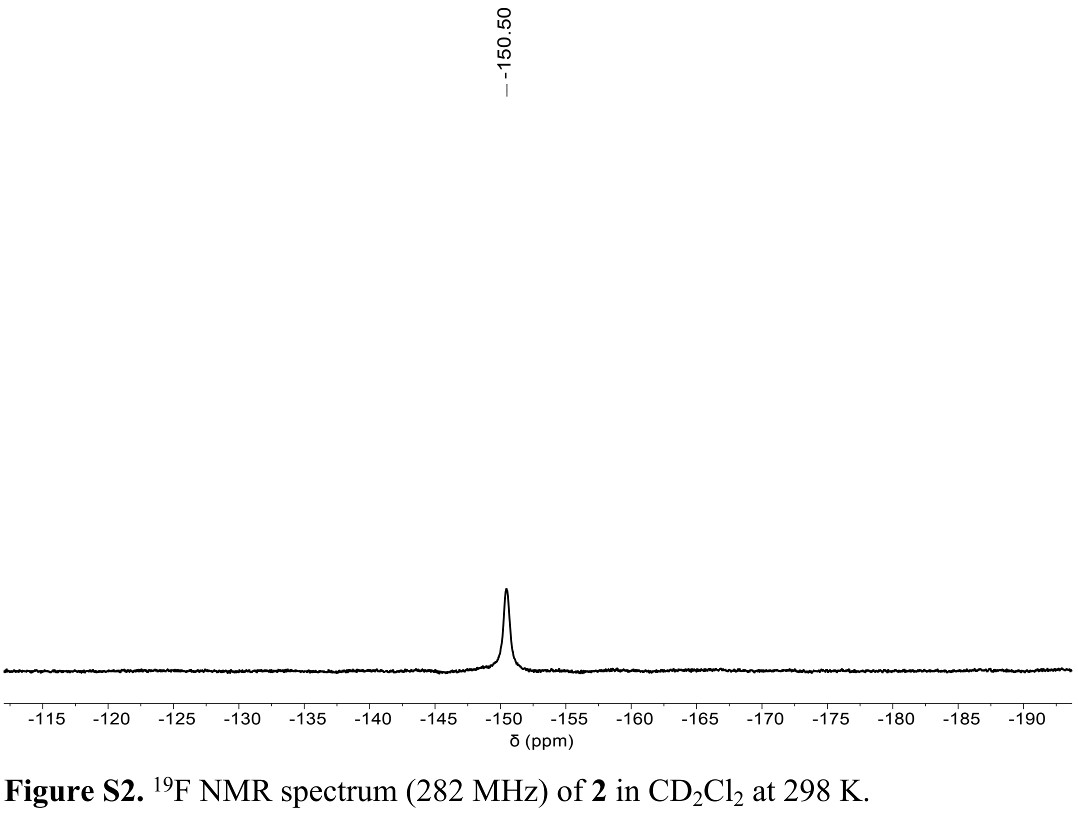


**Figure S2.** $^{19}F$ NMR spectrum (282 MHz) of **2** in $CD_2Cl_2$ at 298 K.

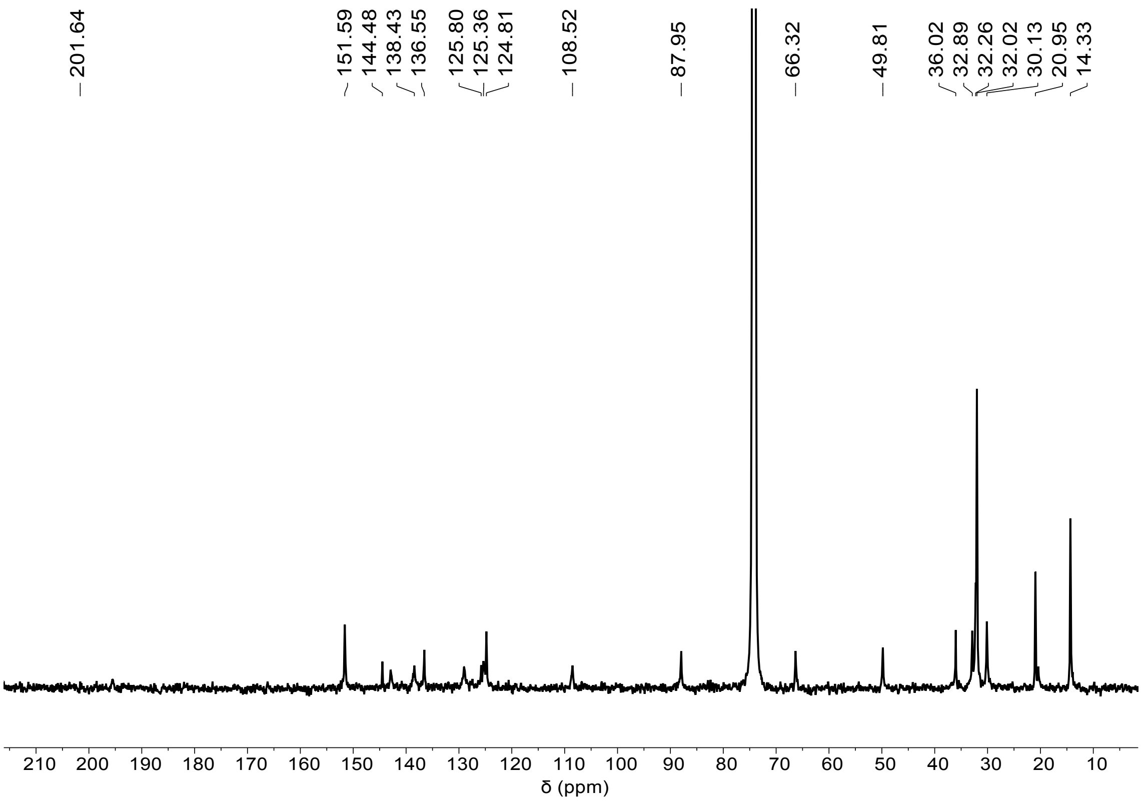


**Figure S3.** $^{13}$C NMR spectrum (75 MHz) of **2** in $C_2D_2Cl_4$ at 298 K.

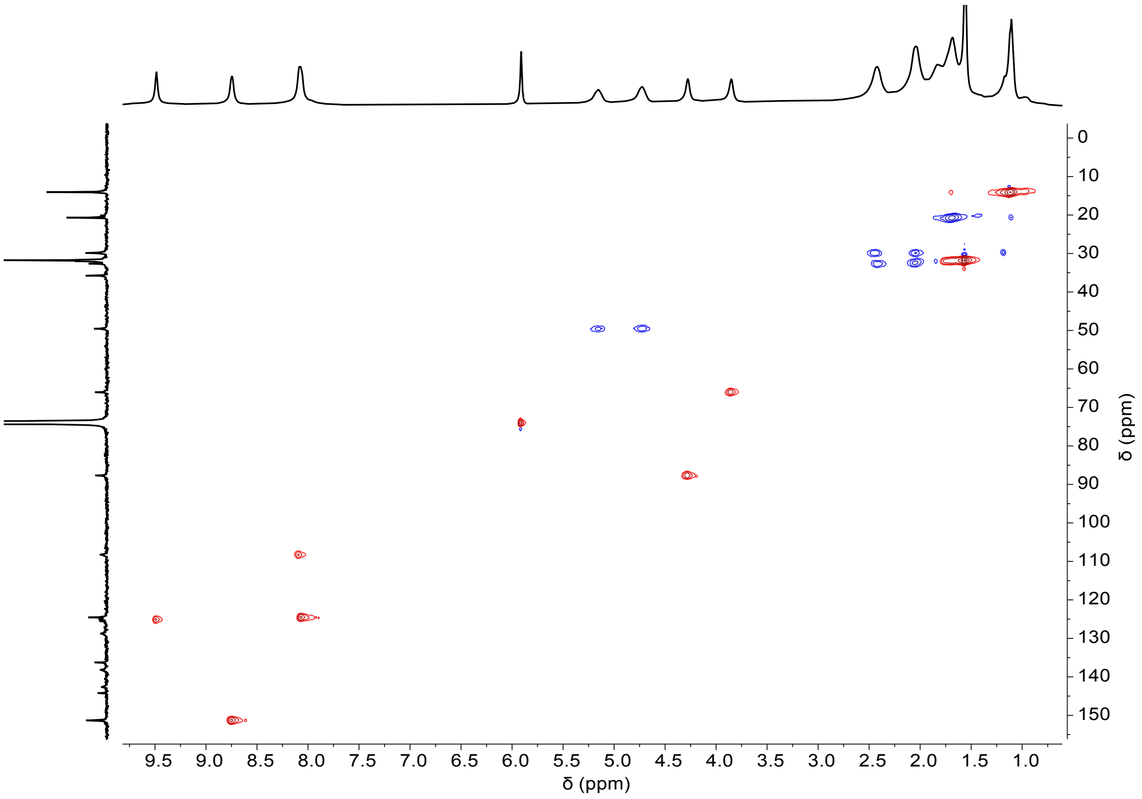


**Figure S4.** $^{1}$H-$^{13}$C HSQC NMR spectrum (300 MHz) of **2** in $C_2D_2Cl_4$ at 298 K.

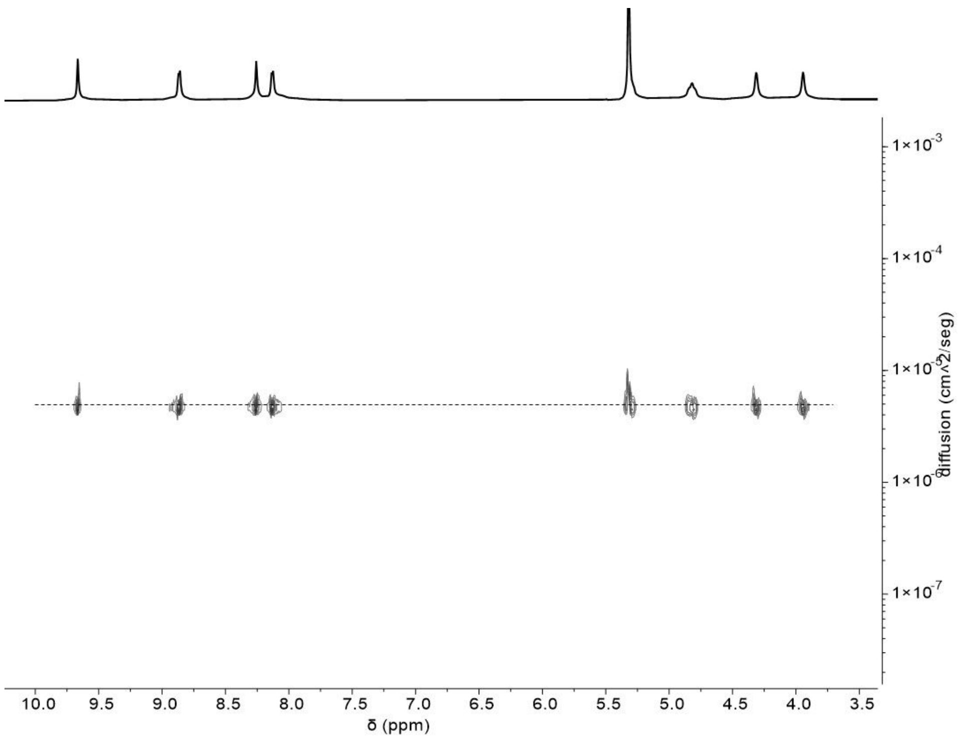


**Figure S5.** DOSY NMR spectrum (500 MHz, 5 mM) of **2** in $CD_2Cl_2$ at 298 K.

## 2. Photophysical properties

### 2.1. UV-visible absorption spectra

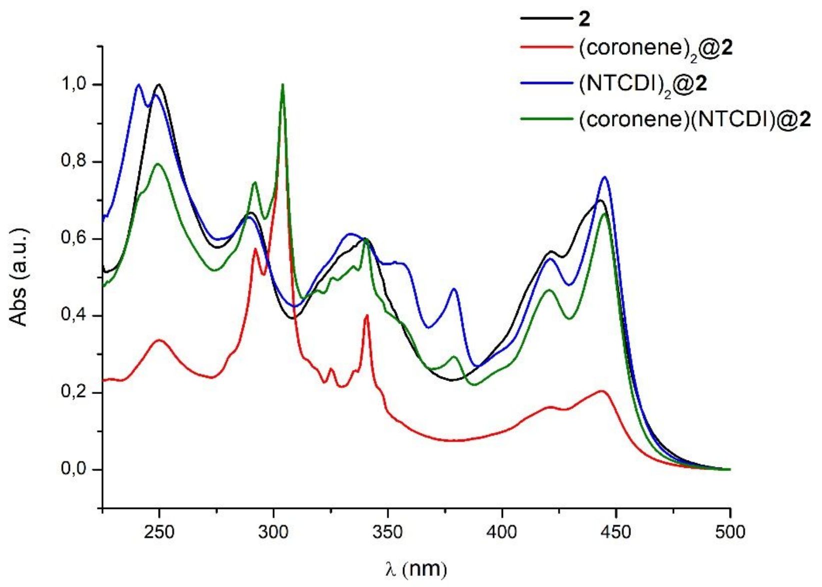


**Figure S6.** UV-visible absorption spectra of complexes **2**, (coronene)$_2$@**2**, (NTCDI)$_2$@**2** and (coronene)(NTCDI)@**2**, recorded in $CH_2Cl_2$ at a concentration of $10^{-5}$ M, under aerobic conditions at room temperature.

### 2.2. Emission spectra

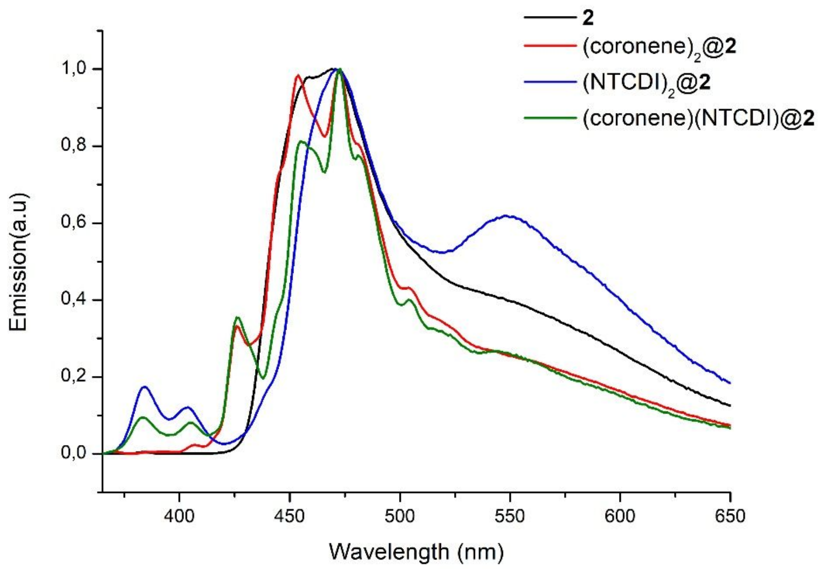


**Figure S7.** Emission spectra of complexes **2**, (coronene)$_2$@**2**, (NTCDI)$_2$@**2** and (coronene)(NTCDI)@**2**, at 345 nm in $CH_2Cl_2$ at a concentration of $10^{-5}$ M, under aerobic conditions at room temperature.

## 3. $^{1}$H NMR titration experiments

The recognition capability of complex **2** (host) were studied by $^{1}$H NMR titration experiments, by adding increasing amounts of pyrene, triphenylene, perylene, coronene or N,N'-dimethyl-naphthalenetetracarboxy diimide (NTCDI) (guest) to a solution of complex **2**. The experiment was carried out in $CD_2Cl_2$, at constant concentrations of the host (1 mM). Two solutions were prepared: solution A (only containing host at 1 mM) and solution B (containing host at 1 mM and guest at different mM). The addition of increasing amounts of solution B to solution A produced perturbations on the signal due to the proton of the pyrene, quinoxaline, or bipyridine core of the host. The association constants were calculated by nonlinear least-square analysis, by using the BindFitv0.5 program.

### Titration of 2 with pyrene

**Table 1.** Data values from the titration study of **2** with pyrene

| **[2]** M | [pyrene] M | $\delta c_H$ | $\delta c_H$ | equiv. pyrene |
|---|---|---|---|---|
| 0,00105491 | 0 | 9,67 | 8,86 | 0 |
| 0,00105491 | 0,00105741 | 9,66 | 8,86 | 2,5 |
| 0,00105491 | 0,00209409 | 9,65 | 8,86 | 3,9 |
| 0,00105491 | 0,00410764 | 9,65 | 8,87 | 5,6 |
| 0,00105491 | 0,0060452 | 9,64 | 8,87 | 7,0 |
| 0,00105491 | 0,00970896 | 9,63 | 8,88 | 9,6 |
| 0,00105491 | 0,01311561 | 9,62 | 8,88 | 13,7 |
| 0,00105491 | 0,01925875 | 9,61 | 8,88 | 19,1 |
| 0,00105491 | 0,02464581 | 9,6 | 8,88 | 22,9 |
| 0,00105491 | 0,03158829 | 9,58 | 8,89 | 34,1 |
| 0,00105491 | 0,03920452 | 9,56 | 8,89 | 38,6 |
| 0,00105491 | 0,04679935 | 9,55 | 8,89 | 48,0 |

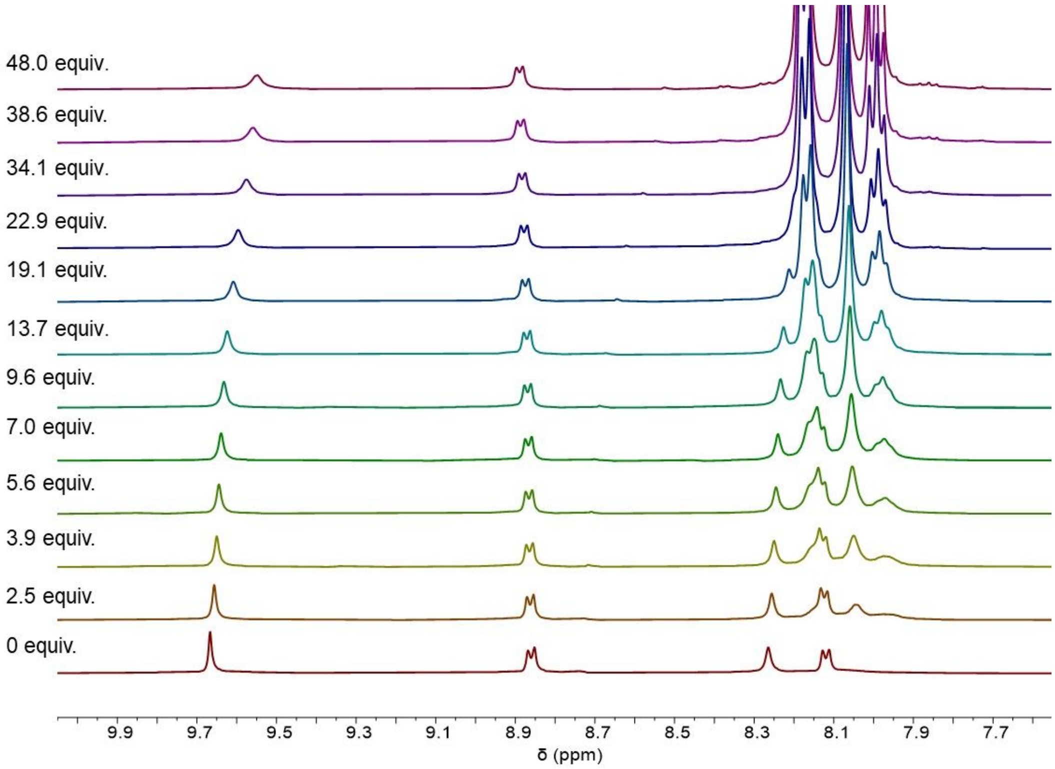


**Figure S8**. Selected region and spectra (400 MHz, $CD_2Cl_2$, 298 K) of the titration of complex **2** with pyrene.

**Titration of 2 with triphenylene**

**Table 2.** Data values from the titration study of **2** with triphenylene

| **[2]** M | [triphenylene] M | δc$_H$ | δc$_H$ | equiv. triphenylene |
|---|---|---|---|---|
| 0,0010264 | 0 | 9,67 | 8,27 | 0 |
| 0,0010264 | 0,00035205 | 9,66 | 8,26 | 0,3 |
| 0,0010264 | 0,00069719 | 9,66 | 8,26 | 0,7 |
| 0,0010264 | 0,00136757 | 9,65 | 8,25 | 1,3 |
| 0,0010264 | 0,00201266 | 9,64 | 8,25 | 1,9 |
| 0,0010264 | 0,00323245 | 9,62 | 8,23 | 3,1 |
| 0,0010264 | 0,00436664 | 9,61 | 8,22 | 4,2 |
| 0,0010264 | 0,00641191 | 9,59 | 8,21 | 6,2 |
| 0,0010264 | 0,00820545 | 9,58 | 8,19 | 8,0 |
| 0,0010264 | 0,01120287 | 9,55 | 8,17 | 11,0 |
| 0,0010264 | 0,01360821 | 9,51 | 8,14 | 13,2 |

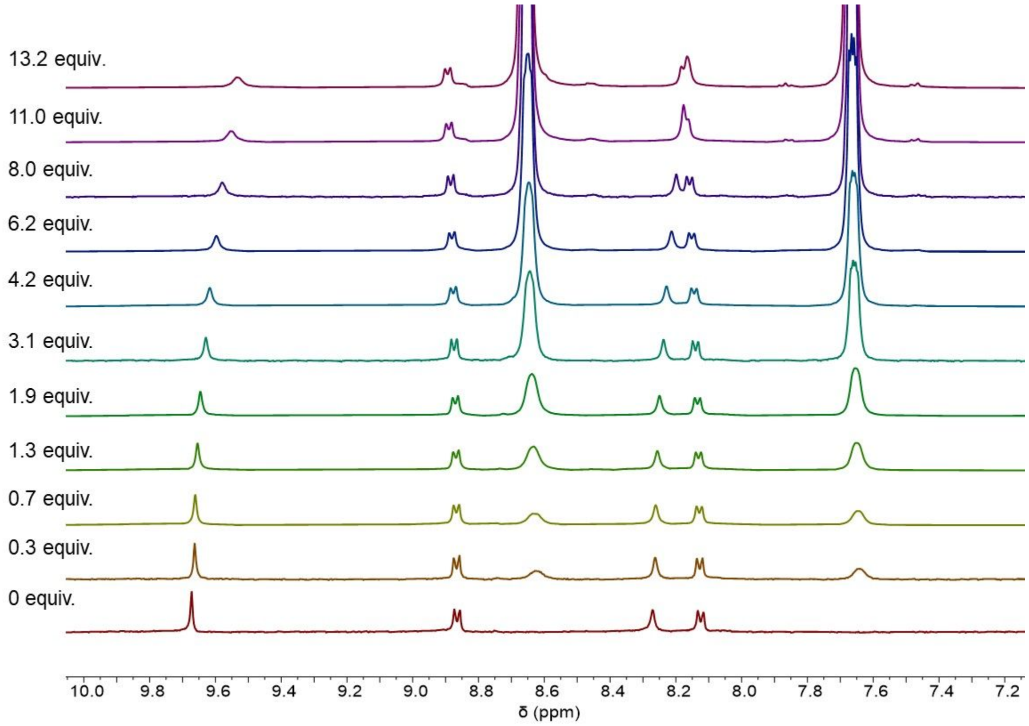


**Figure S9**. Selected region and spectra (400 MHz, $CD_2Cl_2$, 298 K) of the titration of complex **2** with triphenylene.

## Titration of 2 with perylene

**Table 3.** Data values from the titration study of **2** with perylene

| **[2]** M | [perylene] M | δc$_H$ | δc$_H$ | equiv. perylene |
|---|---|---|---|---|
| 0,00101215 | 0 | 9,67 | 8,26 | 0 |
| 0,00101215 | 0,00024291 | 9,67 | 8,26 | 0,2 |
| 0,00101215 | 0,00040486 | 9,66 | 8,26 | 0,4 |
| 0,00101215 | 0,00087045 | 9,66 | 8,25 | 0,9 |
| 0,00101215 | 0,00115385 | 9,65 | 8,25 | 1,1 |
| 0,00101215 | 0,00222672 | 9,64 | 8,24 | 2,2 |
| 0,00101215 | 0,00495951 | 9,62 | 8,21 | 4,9 |
| 0,00101215 | 0,00860324 | 9,58 | 8,18 | 8,5 |
| 0,00101215 | 0,01265182 | 9,56 | 8,18 | 12,5 |
| 0,00101215 | 0,01356275 | 9,56 | 8,18 | 13,4 |

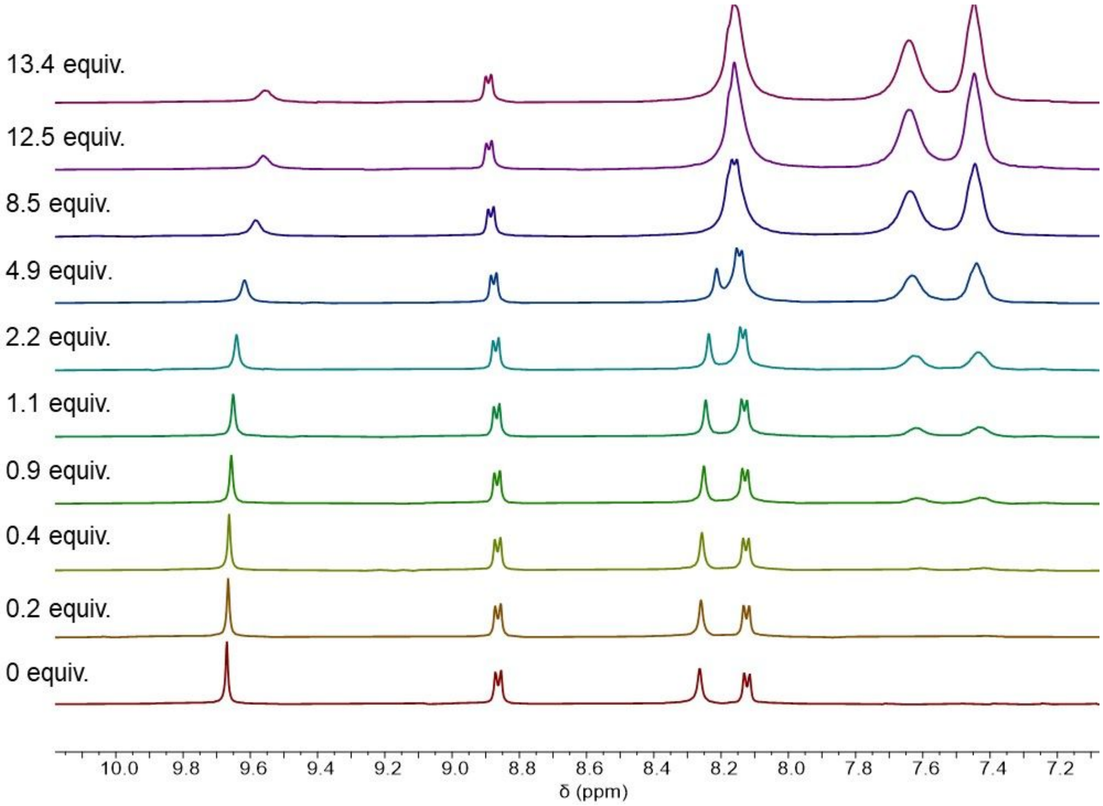


**Figure S10**. Selected region and spectra (400 MHz, $CD_2Cl_2$, 298 K) of the titration of complex **2** with perylene.

## Titration of 2 with coronene

**Table 4.** Data values from the titration study of **2** with coronene

| **[2]** M | [coronene] M | $\delta c_H$ | $\delta c_H$ | $\delta c_H$ | $\delta c_H$ | equiv. coronene |
|---|---|---|---|---|---|---|
| 0,00101215 | 0 | 9,67 | 8,86 | 8,26 | 8,12 | 0 |
| 0,00101215 | 0,00026113 | 9,65 | 8,87 | 8,24 | 8,13 | 0,25 |
| 0,00101215 | 0,00051222 | 9,63 | 8,88 | 8,22 | 8,14 | 0,5 |
| 0,00101215 | 0,0009865 | 9,58 | 8,89 | 8,17 | 8,17 | 1,0 |
| 0,00101215 | 0,00183694 | 9,48 | 8,92 | 8,06 | 8,22 | 1,8 |
| 0,00101215 | 0,00291327 | 9,32 | 8,96 | 7,89 | 8,29 | 2,9 |
| 0,00101215 | 0,00406933 | 9,15 | 9 | 7,71 | 8,36 | 4,0 |
| 0,00101215 | 0,00499417 | 9,06 | 9,03 | 7,59 | 8,4 | 4,9 |
| 0,00101215 | 0,00591902 | 8,93 | 9,05 | 7,49 | 8,45 | 5,8 |
| 0,00101215 | 0,0066589 | 8,97 | 9,06 | 7,45 | 8,46 | 6,6 |

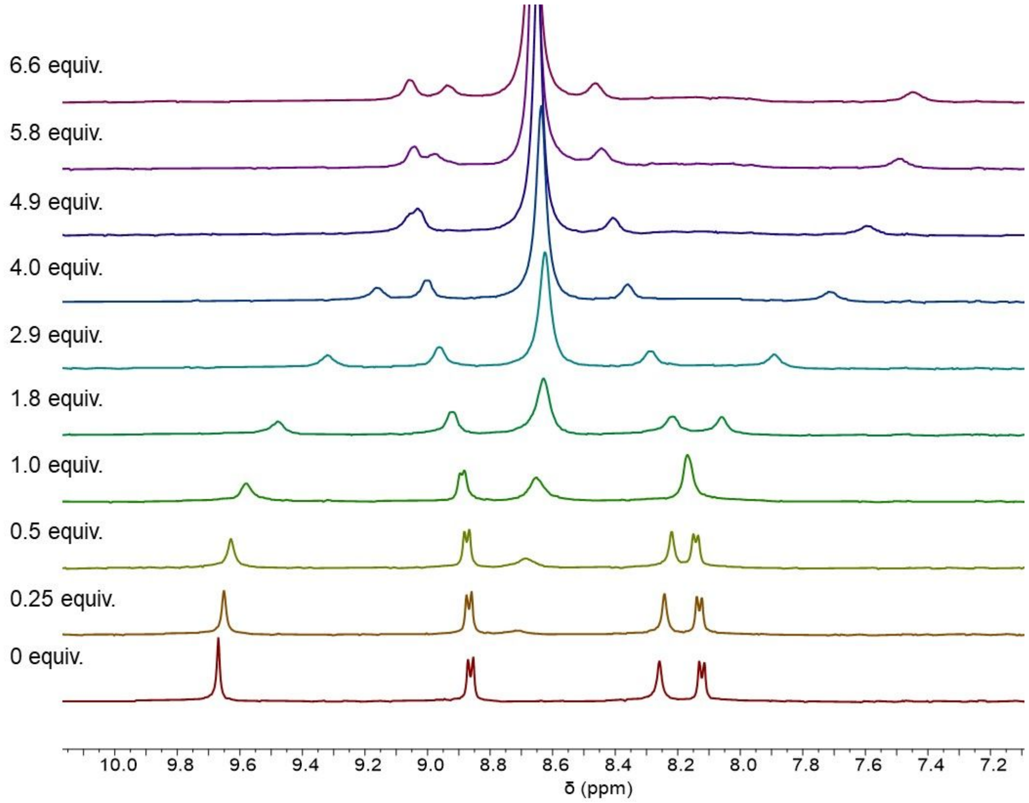


**Figure S11**. Selected region and spectra (400 MHz, $CD_2Cl_2$, 298 K) of the titration of complex **2** with coronene.

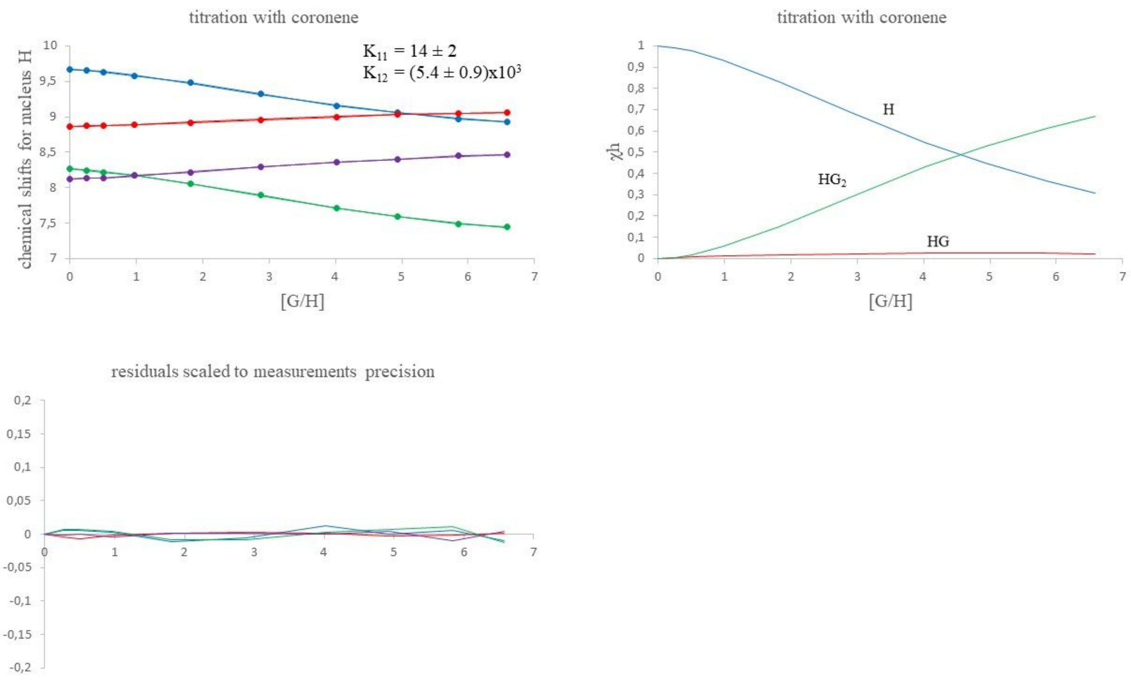


**Figure S12.** Non-linear least-squares fitting of the chemical shift changes of H during titration experiments of **2** with coronene. The Figure on the left represents the speciation profiles.

## Titration of 2 with NTCDI

**Table 5.** Data values from the titration study of **2** with NTCDI

| **[2]** M | [NTCDI] M | δc$_H$ | δc$_H$ | δc$_H$ | δc$_H$ | equiv. NTCDI |
|---|---|---|---|---|---|---|
| 0,00101215 | 0 | 9,67 | 8,86 | 8,26 | 8,12 | 0 |
| 0,00101215 | 0,00041826 | 9,65 | 8,86 | 8,25 | 8,13 | 0,4 |
| 0,00101215 | 0,00080554 | 9,63 | 8,87 | 8,25 | 8,13 | 0,8 |
| 0,00101215 | 0,00149996 | 9,57 | 8,88 | 8,23 | 8,14 | 1,5 |
| 0,00101215 | 0,00210479 | 9,51 | 8,89 | 8,21 | 8,16 | 2,1 |
| 0,00101215 | 0,00310707 | 9,4 | 8,9 | 8,18 | 8,2 | 3,1 |
| 0,00101215 | 0,00390375 | 9,33 | 8,92 | 8,16 | 8,21 | 3,8 |
| 0,00101215 | 0,00483322 | 9,27 | 8,93 | 8,15 | 8,21 | 4,8 |
| 0,00101215 | 0,00554398 | 9,24 | 8,93 | 8,14 | 8,21 | 5,5 |
| 0,00101215 | 0,0062668 | 9,24 | 8,93 | 8,14 | 8,21 | 6,2 |
| 0,00101215 | 0,006817 | 9,24 | 8,93 | 8,14 | 8,21 | 6,7 |

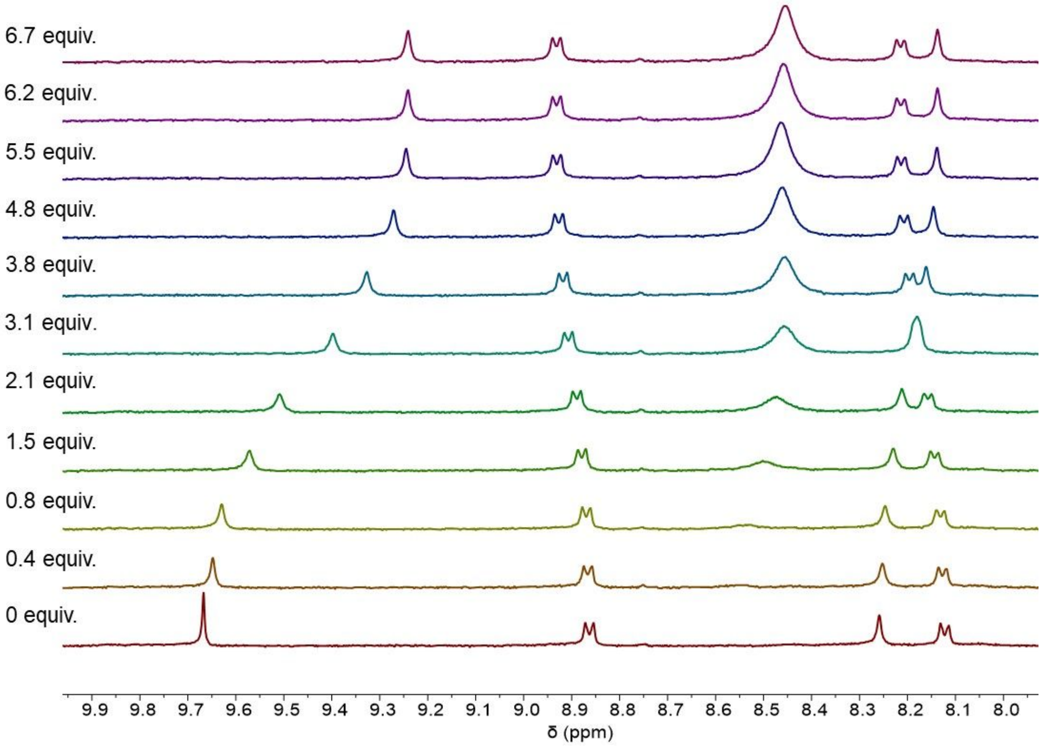


**Figure S13**. Selected region and spectra (400 MHz, $CD_2Cl_2$, 298 K) of the titration of complex **2** with NTCDI.

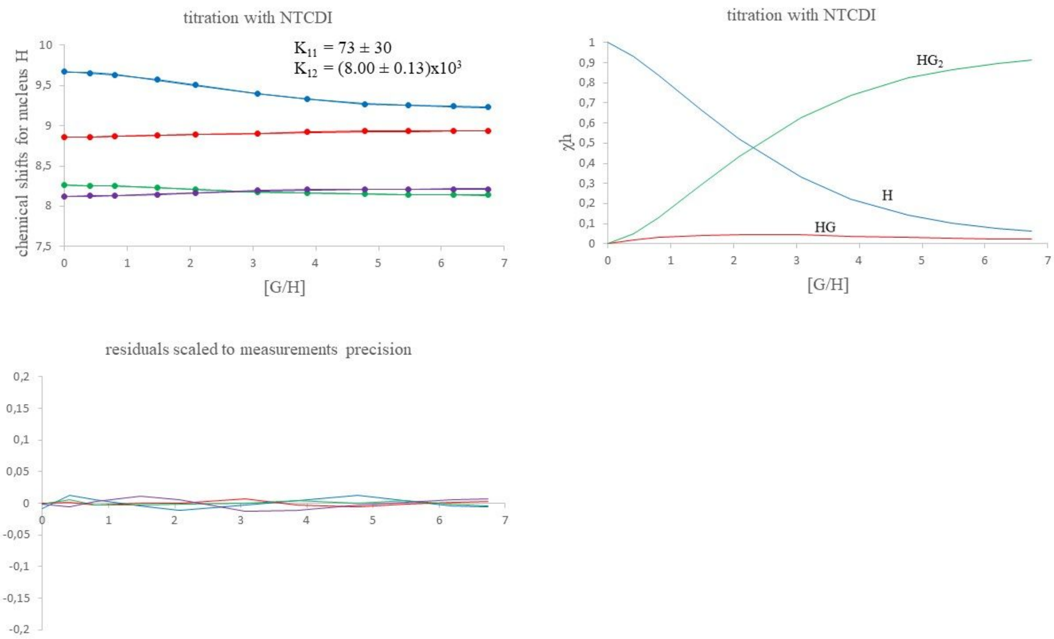


**Figure S14.**Non-linear least-squares fitting of the chemical shift changes of H during titration experiments of **2** with NTCDI. The Figure on the left represents the speciation profiles.

## 4. Variable-temperature $^1$H NMR experiments

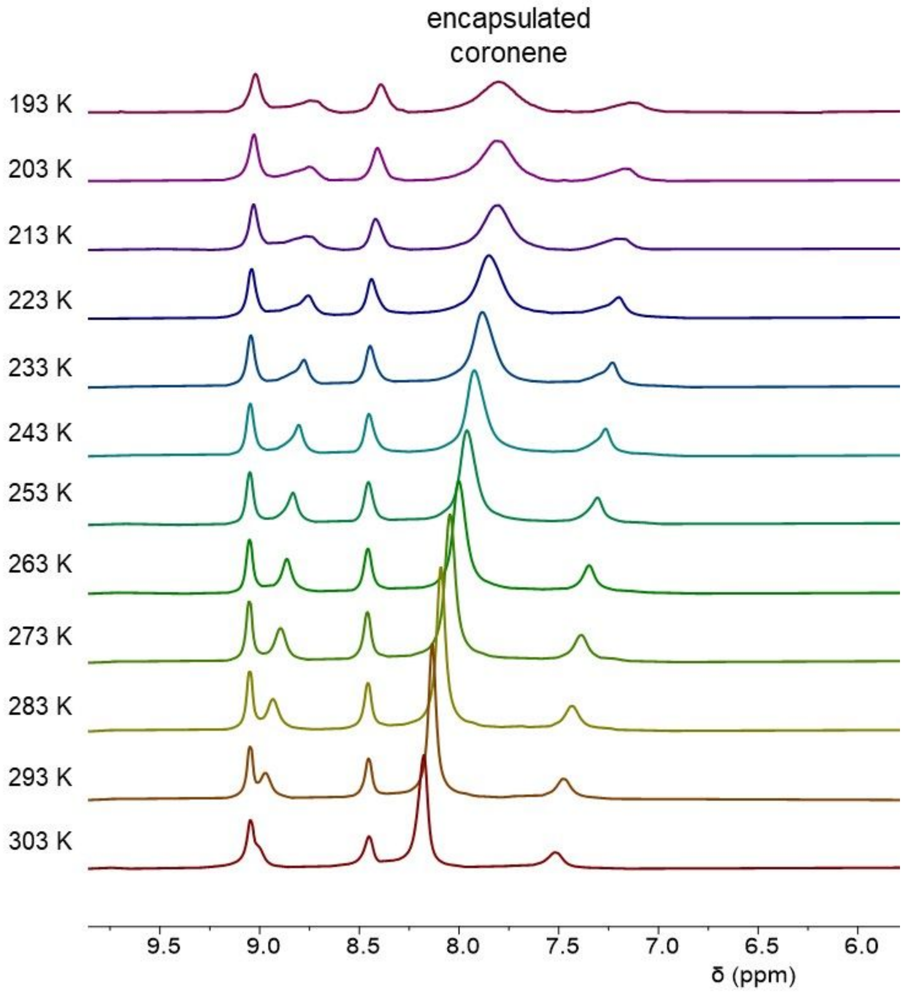


**Figure S15.** Selected region of the $^1$H NMR spectra (500 MHz) of complex (coronene)$_2$@**2** in $CD_2Cl_2$ at different temperatures.

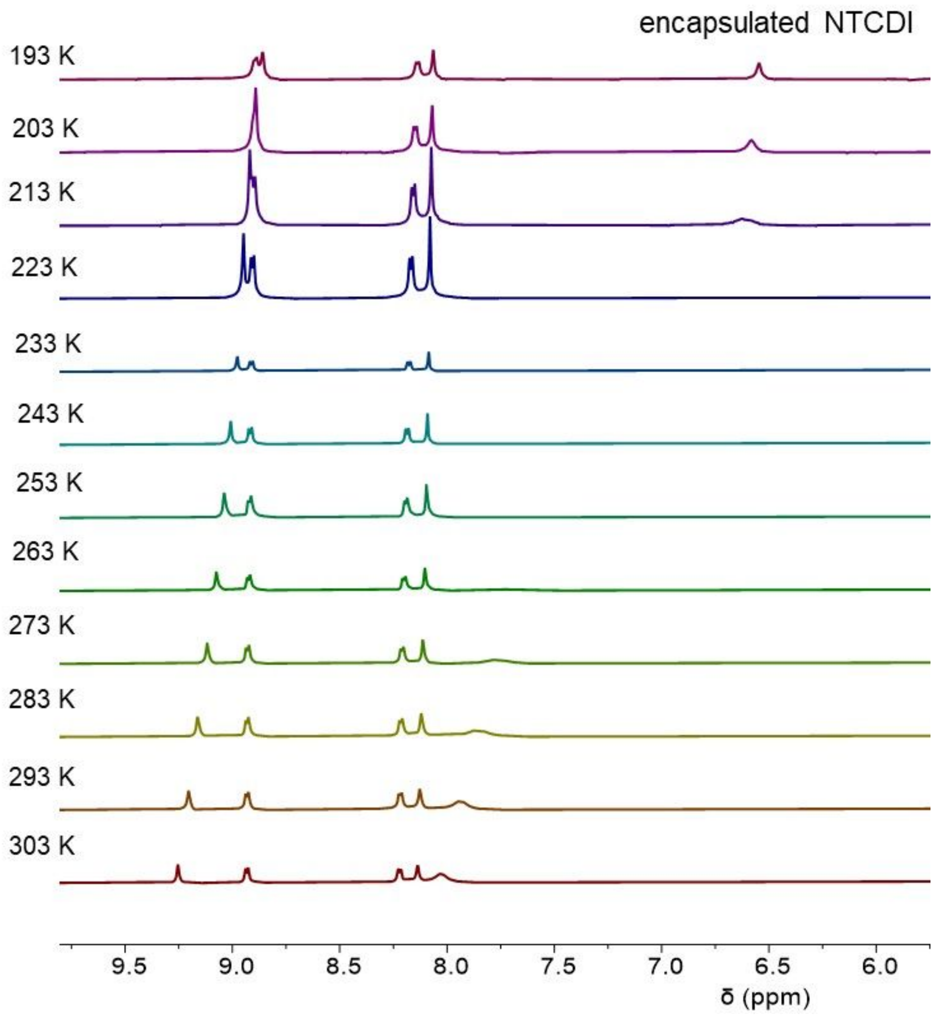


**Figure S16.** Selected region of the $^1$H NMR spectra (500 MHz) of complex $(NTCDI)_2$@**2** in $CD_2Cl_2$ at different temperatures.

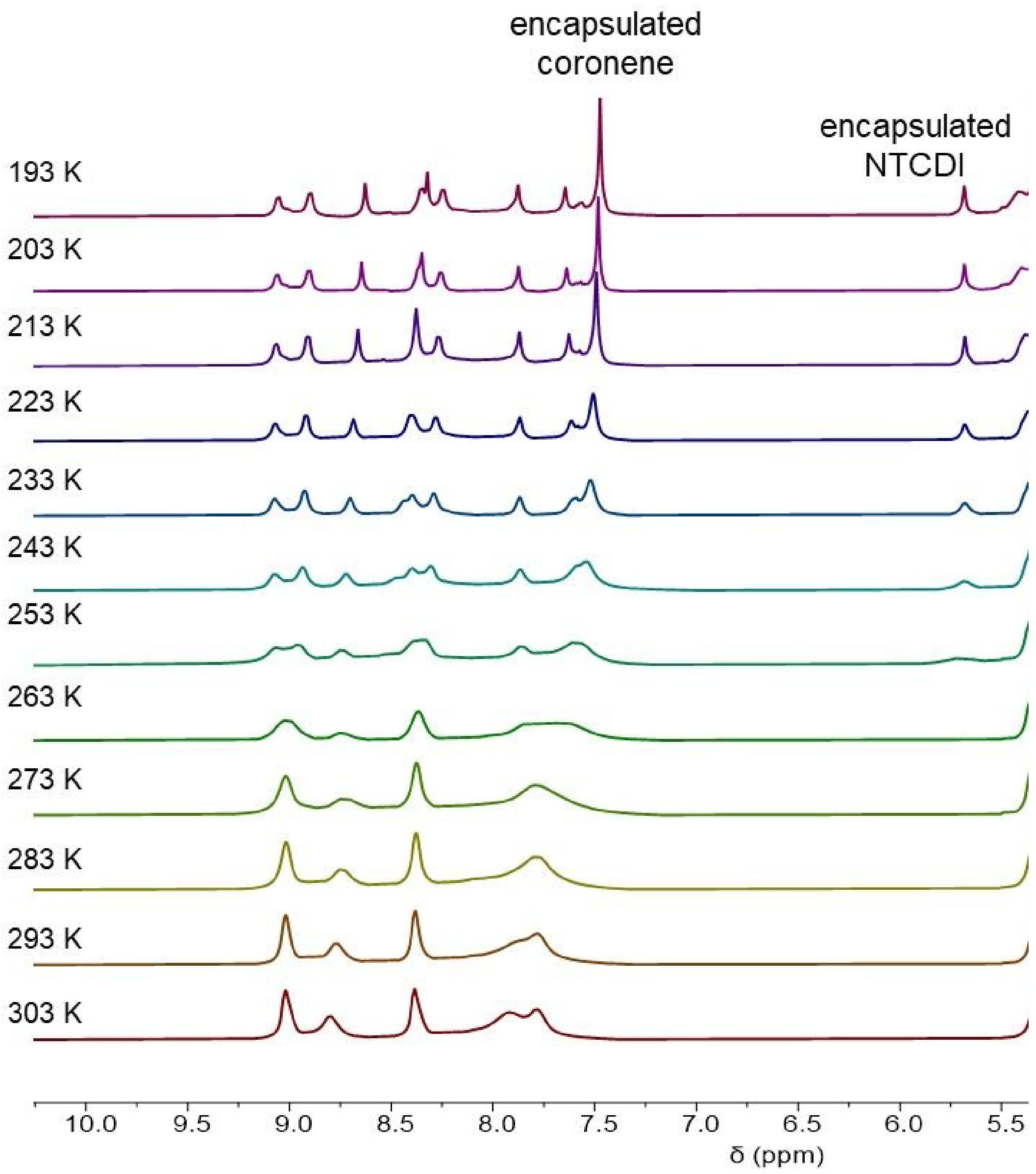


**Figure S17.** Selected region of the $^{1}H$ NMR spectra (500 MHz) of complex (coronene)(NTCDI) @**2** in $CD_2Cl_2$ at different temperatures.

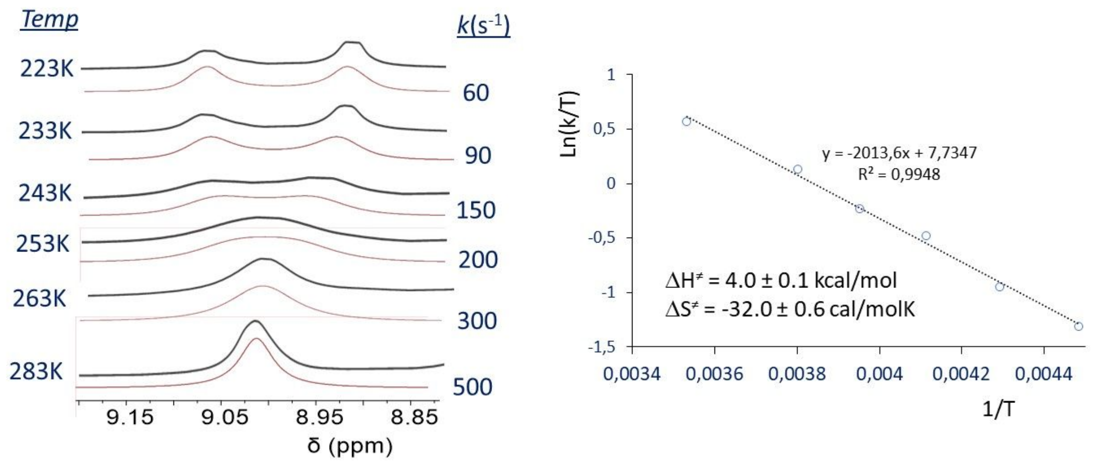


**Figure S18.** Study of the dynamic behavior of (coronene)(NTCDI)@**2** investigated by variable temperature $^{1}$H NMR spectroscopy. All spectra recorded in $CD_2Cl_2$. The determination of the kinetic constants was performed by dynamic $^{1}$H NMR simulations using SpinWorks 4.0. Black traces and red lines represent experimental and simulated data, respectively.

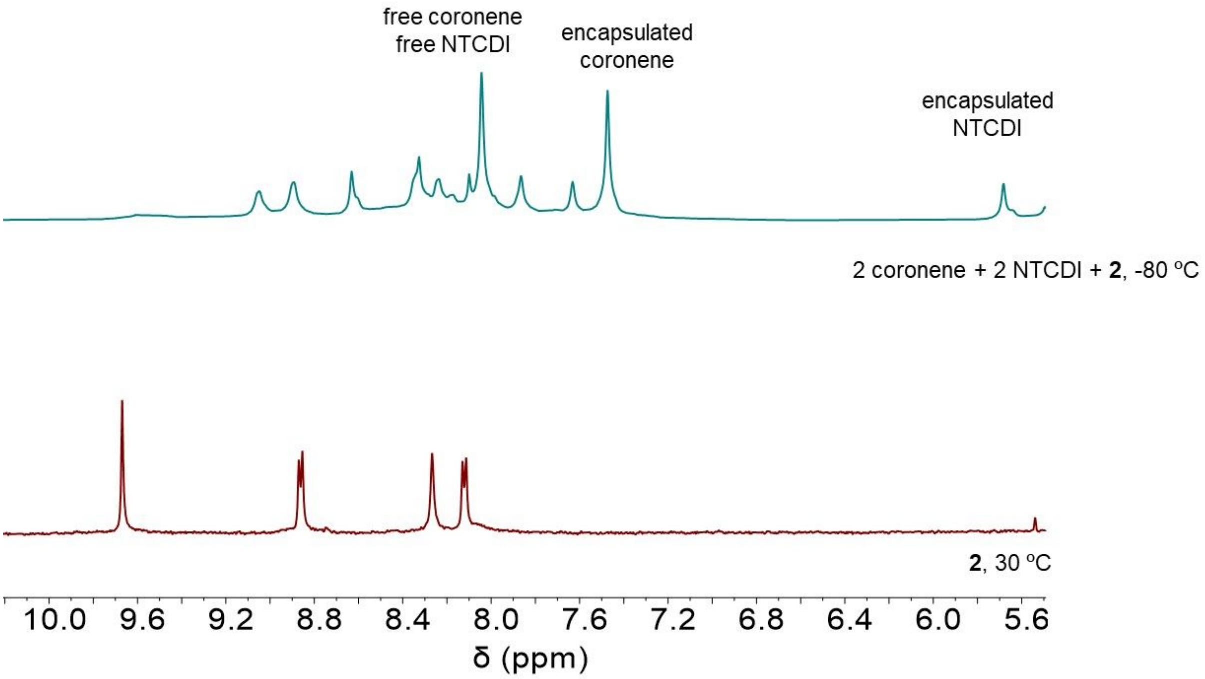


**Figure S19.** From botton to top: aromatic region of the $^{1}$H NMR (500 MHz) spectra ($CD_2Cl_2$) of metallobox **2** at 30 ºC, and 2 eq. of coronene + 2eq. of NTCDI + 1eq. of **2** at -80 ºC.
